# LGBTQ+ Well-Being in Rural US: Barriers and Promotion Opportunities

**DOI:** 10.1093/geroni/igaf122.1728

**Published:** 2025-12-31

**Authors:** Bryce Van Vleet

## Abstract

LGBTQ+ aging adults across geographies have disproportionately poor well-being outcomes compared to aging non-LGBTQ+ adults. However, rural communities pose additional structural challenges for individuals across the LGBTQ+ spectrum. This mixed-methods symposium highlights burgeoning research on understudied, and often under resourced, LGBTQ+ rural adults. Van Vleet and Fuller introduce LGBTQ+ disparities across queer identities in rural areas and find that social support does not buffer discrimination’s effects on mental health. Johnson investigates disproportionate material-need insecurity among diverse, rural Texan LGBTQ+ adults and poses suggestions for investigating LGBTQ+ populations in public datasets. The last two papers investigate barriers to social support and adequate services among LGBTQ+ older adults in rural areas. Perone and Zhou highlight the need for culturally-informed social services and increased need for LGBTQ+ gathering spaces. Bietsch further affirms these findings in rural New England, suggesting LGBTQ+ older adults have disproportionate access to social support and culturally-responsive healthcare. This symposium investigates LGBTQ+ well-being across rural contexts within the United States and offers strategies to promote well-being among adult and aging LGBTQ+ populations. Strategies for researching LGBTQ+ topics and developing interventions in rural areas are also discussed.

